# Medical Association of Central New York

**Published:** 1910-10

**Authors:** C. R. Greenleaf

**Affiliations:** Secretary


### SOCIETY PROCEEDINGS

### Medical Association of Central New York

REPORTED BY C. R. GREENLEAF, M.D., Secretary.

*(Concluded from September.)*

DR. CREVELING: The next paper on the program is by Dr. Robert T. Morris, of New York, "Protective Appendicitis."

DR. ROBERT T. MORRIS, New York: Mr. President, members of the association, in the course of development of any one subject we are apt to elaborate more and more until the proportion is apt to be lost. It is important for us to keep, then, our sense of proportion in order to give relative value to our facts. Some years ago Senn described what he called appendicitis obliterans. Very little attention was given to this subject, although his article was of very great value, as were most of the monographic articles given to the profession by Senn. Senn, however, had not made a detailed study of the subject, but he said that a certain number of cases of appendicitis were ones in which there were frequently repeated minor attacks, the patients were not often confined to the bed, and the appendix on examination was found to be hard and more or less degenerated. He advised removal of these appendices, and believed that they represented apparently one feature of some chronic infection in this region.

The subject passed practically out of mind of the profession, and later Ribbert described about this same condition, finding in a very large proportion of all post mortem examinations in patients or people past the age of fifty that the appendix was degenerating, often represented by a mere string, and he believed that the condition was one occurring in the course of evolution, and representing a normal involution of the appendix. He called these cases of normal involution of the appendix, and expressed the opinion that this condition was a most common cause for appendicitis. He believed that in the course of the degeneration infective processes gained entrance and the features of infective appendicitis as we see them today developed. On taking up a study of this subject I seem to be led to a very different conclusion from the conclusion of Ribbert and of Senn. I seem to find that in these cases the patients are protected against infective appendicitis, and in two ways. The first way is this: in the course of normal involution of the appendix the tissues susceptible of infection are removed, mucosa, sub-mucosa, lymphatic layer, one by one disappear. These are the structures which are most susceptible of infection and which cause the chief disturbance in acute infective appendicitis. These cases I would call cases of protective appendicitis, because the process causes a disappearance of the very structures susceptible of infection. I would further call it protective appendicitis, because there is a constant irritation of these degenerating appendices.

The reason why there is irritation is: there is a gradual replacement of various structures of the appendix by hyperplastic connective tissue. This hyperplastic connective tissue contracts in the appendix, as it does in other parts of the body, and precisely as in other parts of the body it engages nerve filaments, and when it engages nerve filaments in its contracting mass it irritates them precisely as nerve filaments in an amputated stump of an arm or leg. This source of constant irritation calls out a persistent local hyperalgesia. It sets in motion the protective mechanism and machinery of the patient in that region. Therefore, we have two well marked and distinct reasons for the nomenclature which I propose of protective appendicitis for these cases, and they seem to me to represent the most common form of appendicitis.

We can for purposes of classification convenience make today four kinds: first, and most common, what I choose to call at present protective appendicitis. Next most common is the form of intrinsic infection of the appendix. With intrinsic infection of the appendix we have a rapid swelling of the inner coats to the points of compression anemia. This includes the idea of a constriction of the lumen, but we have compression anemia due to rapid swelling of the inner coats within the tight outer sheath. When we have compression of the swollen tissues within the tight outer sheath, bacteria attack the anemia area, which is temporarily infected, and that is why appendicitis, acute infective appendicitis, runs such a very different course from the course of other and similar infections two inches away in the cecum, which can swell freely without giving rise to anemia and leaving foci for bacterial attacks.

The ordinary, most common form of appendicitis is perhaps the one which occurs, with various other causes, after serous infiltration in the tissues of organs. Where tissues are distended with serous infiltrates there is a swelling of the appendix incidentally, and often with a good deal of pain in the vicinity, because of the swelling of the tissues within the tight outer sheath again, but the appendicitis in those cases is an irritative lesion rather than infective, the tissues protect themselves, all structures about are suffering from the same disturbance, the serous infiltration. It occurs often enough with so-called rheumatic inflammation of the appendix. It occurs with loose kidney. That is one of the irritative forms of appendicitis. The other type is the infection by the extrinsic means, where it extends from the peritoneum and swelling occurs so slowly that we do not have the incident of acute compression anemia, not very often. So there are two irritative forms of appendicitis and two infective forms, and this irritative form, which is most common, which is generally overlooked, I will speak of as protective appendicitis, believing that it actually protects the patient against infection.

These are the patients who go the rounds of the profession asking whether they have appendicitis or not, and if they see a sufficient number of doctors they will in the end have a sufficient number of opinions. These patients are particularly apt not to go to bed. Why ? Because there is no irritative lesion at work, and it keeps them just active enough so that, in the words of Oliver Wendell Holmes, they accomplish very much more than well men. We find as a result of this protective appendicitis two sets of symptoms. A very common one is a feeling of burning in the region of the appendix, occasionally coming, occasionally going, somtimes with an actual pain, but as a rule giving little more than common discomfort. The patient is inclined to press over the appendix with his hand; he makes his own diagnosis of appendicitis in these days, and really does not have, however, at any time a very severe attack of pain. The other set of symptoms depends upon irritation of the intimate ganglia of the bowel wall, of the plexus.

These plexus are irritated and the bowel function disturbed, so that the patient suffers from what is called chronic intestinal indigestion, and a very important part of all of the cases of so-called chronic intestinal indigestion are dependent upon the presence of an appendix which is undergoing this form of degeneration. (Dr. Morris produced certain photographs and specimens.) This photograph, the single specimen, is one in which the appendix looked quite normal when it was removed, and excepting for the subjective history I presume it would not have been removed. In cases of this sort surgeons have many times opened the abdomen, have seen what they believed to be a normal appendix, have felt that they made a mistake in diagnosis, and sometimes have closed the abdomen, and the patient has gone on suffering, when if they had believed in removing a normal appendix the patient would have been cured. So far as that point is concerned, I do not believe in removing a normal appendix where it happens to appear in the wound, for the reason that the area is protected by these local conditions. On that ground I am opposed to the idea of removing the normal appendix. But this appendix was like a normal one, and one would need the subjective history in connection to make the diagnosis, but on section, longitudinal section, we find the inner structures are all replaced by hyperplastic tissue, and no lumen. That patient had been suffering for years, had gone the rounds of the hospitals and baths in this country and in Europe, and without getting relief and that is true of both of the patients from whom these two specimens were removed. Both of these patients, one a retired naval officer and one a woman, who had been quite important in the fashionable world in New York, had given up trying to enjoy health, had been the rounds of various sanitariums, both in this country and in Europe, because of their chronic intestinal dyspepsia, and both were entirely cured immediately upon removal of what would seem to be very unimportant appendix remains, but these remains did not contain nerve filaments which were engaged in hyperplastic, sclerotic connective tissue, which irritated constantly their intimate ganglia of the dowel wall. Here is a case in which about one half of the appendix has undergone fibroid degeneration and the rest is practically normal (showing specimen). These show for themselves very well. Here is one in which even the peritoneum has disappeared, and gradually the most of the appendix disappears. I am sorry that I did not have time to put these in fresh bottles. I am just back from my vacation and got out these specimens hurriedly last night. Here is one in which we have alternative nodes and internodes of fibroid degeneration and the mucous remains. Here are two of the specimens shown in one of the photographs. Here is one shown in the first photograph, looking quite like a normal appendix, but without lumen remains. There is no need for making further remarks on the subject primarily, because I have now presented the salient features, and I will be glad to answer questions relating to the subject, which may bring out further facts which members of the Association may wish.

Dr. Miller: I think there are many here who are familiar with the work which Dr. Morris has done with the subject which he has presented, and know there is none more able to present the subject than Dr. Morris. The thing that is troubling the ordinary physician is the matter of diagnosis, and the ability to eliminate disease of the appendix from disease possibly of other portions of the body. Some of us know that Dr. Morris has presented views which he has worked out and that we believe in implicitly, and probably there are many here who would be glad to hear from Dr. Morris on the subject of diagnosis, and the matter of differentiating between the disease of the appendix, or disease in the region of the cecum, and those, perhaps, of the organ of generation in the female, at least.

Dr. Johnson: There are a few points about why the appendix degenerates that have been of interest to me. I have heard Dr. Morris once before tell the points in the pathology. As long ago as 1870, Darwin said that the appendix was a vestigial structure and implied that it was born old, that it was in process of elimination, and that the point in embryology would prove that, as in other vestigial structures, it is out of proportion in the embryo. The embryonic appendix is larger, related to the rest of the intestinal tract, than it is in the adult, so that the appendix may be said to be born old and be senile at the birth of the individual carrying it. Because of this senility it is liable to trouble. Vestigial structures are poorly nourished, they are of no use in the general economy, and the general economy recognises that and starves them so they have not the resisting power that useful organs have. One point that supports that is the early date at which cancer of the appendix appears. More than half of the cases of cancer of the appendix appear before thirty years of age, which is quite contrary to the general history of cancer of the organs of the body. The appendix is a true example of that thing, because more than half of the cases of malignancy come before the thirtieth year. When I saw the title of the doctor's paper, "Protective Appendicitis," it was a new one to me. I did not get that meaning; I did not get his meaning, but the other point that he has got I like very well, but whether it is really protective, I will have to think of that another year.

The President: It appears to me, gentlemen, that Dr. Morris ought not to come all the way up from New York and deliver to us this interesting talk without having heard from some of you gentlemen, who are so well able and whose experience in this line of work surely leads you to that particular kind of work, that you ought not to let the subject drop without at least endorsing or disagreeing with the views he has expressed.

Dr. Sears: I think there is a much larger group of those cases than we ordinarily take into consideration; that in many cases where we hesitate about removing the appendix it would perhaps be wiser to do so. I know of one case I observed in which I found just a small abscess in the tip of the appendix. There is no question in my mind at all but what this was the cause of the woman's whole trouble. One question I want to ask Dr. Morris, and that is regarding patients complaining of a feeling of pain when walking up hill or upstairs, particularly in walking up hill. A number of my patients have complained that they were sore after walking up hill.

Dr. Jones, Rochester: I think Dr. Morris's idea is a very good one, and I think it applies to a very annoying class of cases; I refer to chronic mucus collitis. I do not mean to always advocate the removal of the appendix, but there are many cases we are almost absolutely powerless to cope with, which I think can be traced to this cause. The cases are rather few, but all too many, of mucus collitis. You all know what it means, and among other things it means a chronic case for probably a lifetime, and the symptoms are an intractible diarrhea, with pain, mucus discharges later on, and disorganised nervous and mental system, and almost a useless being. There may be many other causes besides an appendicitis. I wish Dr. Morris would tell us how often an appendicitis may be related to the chronic, intractible, incurable, exasperating, mucus collitis.

Dr. Wallace: It seems to me that any attack of appendicitis is a protective appendicitis if the patient recovers from it. In other words, I think that the first attack of appendicitis is very much apt to be the most dangerous that the patient ever has, and I would like to ask Dr. Morris if possibly the matter of accident, for instance, the character of the infection, or the possibility of the presence of a fecal concretion, would not enter more largely into the question of making the attack of peritonitis a protective rather than an infectious peritonitis, rather than the simple, chronic, slow fibroid inflammation. I would like to bring up another point. Doesn't this same question of the extra danger of the first attack of appendicitis, the unusual danger, make appendicitis in children very much more serious than in the adult? Because in most cases of adults with appendicitis have they not had some previous inflammation which is to a certain extent a protective peritonitis or appendicitis.

Dr. Morris: In regard to Dr. Miller's point of a diagnosis, there are two or three points which make it very easy for us to make a diagnosis, as a rule, of protective appendicitis. If we make pressure on the right group of lumbar ganglia, an inch and a half about to the right of the navel, and close down upon the spinal column, if we make pressure at that point and find this group of lumbar ganglia hypersensitive without any question, and if we find that on the corresponding left side there is no hypersensitiveness of the lumbar ganglia, look to the appendix regularly for the source of your trouble. If both groups of lumbar ganglia are hypersensitive, right and left, look to your pelvis for the source of trouble. This will give us the diagnosis regularly. It is of importance in making a diagnosis of acute appendicitis at times, but McBurney's point is of so much greater value that I have not laid much stress upon this particular point in cases of acute appendicitis, but in making the diagnosis of protective appendicitis there let us find the right group of lumbar ganglia hypersensitive, singly and alone. There are other points, but it would be confusing to bring them in, and we do not need any other.

Dr. Johnson's point, his calling attention to the presence of cancer early in the appendix, and showing that we are born with the appendix old, and referring to its vestigial origin, is quite in line, I believe, with the common belief of the day—not with the common belief, but with the scientific belief of the day. Dr. Johnson is not so sure about the nomenclature of protective appendicitis. Now, my friend, Dr. Lee of New York, said precisely the same thing, has for sometime, and not long ago came into the office with an appendix in a bottle and said: "Here, Morris, is one of your cases of protective appendicitis; I have been watching this patient for a year or two; she has had grumbling attacks, and she has had an acute one." I took it to Dr. Brooks, who made microscopical sections. This was acute; there was pus. Dr. Brooks made microscopical sections and found none of the evidences of fibroid degeneration in that appendix. So that it wasn't one of my cases of fibroid degeneration of the appendix. It was probably,—had been a mucus inclusion case. They will grumble on for a year or so before they have an explosion very frequently.

Dr. Sear's point: He spoke of a case in which there was a little mucus at the tip of the appendix, which was close to the ovary, and that probably was a mucus inclusion case. In patients who speak of tenderness in the appendix region after walking up hill, you will find a good many of those cases in the class of protective appendicitis.

Dr. Johnson says he hasn't heard the term "Protective Appendicitis" previously. This is a new nomenclature which I have just proposed. I proposed it at Budapest last month for the first time, and it is now a question if we shall accept the nomenclature or not. It will depend upon the members of the profession. I merely make the proposal.

Dr. Jones asked in how many cases of mucus collitis is a fibroid degeneration of the appendix a factor? Now and then we find a case in which that is the precipitating factor. Cases of mucus collitis, it seems to me, belong to the neurotic temperaments; it seems to me that they belong to patients who are apt to have a psychosis. They are cases in which the collitis is merely a symptom, like a cough or a sneeze, and just as many causes will make one cough or sneeze, so many peripheral irritations will cause a mucus collitis as precipitating causes. In cases in which the neurotic habit has not become established, if we remove the peripheral irritation we may very often remove the symptoms of mucus collitis very markedly, and I have had two or three cases in which a fibroid degeneration of the appendix was the precipitating cause in the case, and the patients became practically well after its removal. I have had others in which I told the patient and their friends that removal of this condition was going to cure their symptoms, and it didn't. That was some years ago. I am very careful about telling them that now. There are more cases of eye-strain acting as the precipitating factor in mucus collitis than there are cases of fibroid degeneration of the appendix. There are perhaps more cases of loose kidney acting as the precipitating factor than there are cases of fibroid degeneration of the appendix, but a few of all of these peripheral irritations serve as the precipitating factor in cases of mucus collitis in a class of cases prone to that form of demonstration, that form of symptom which represents a cough or a sneeze in other parts of the body apparently.

Dr. Wallace says that any attack of appendicitis is protective. That is true. We are apt to have more or less destruction of the parts susceptible to infection in any acute attack of appendicitis, but there are very many cases in which a destruction of a part of the appendix in an acute infective attack leaves a mucus inclusion, and then we have endless trouble and great distress and subsequent attacks. In about 12 per cent. of all cases of appendicitis there are concretions, and if the patient recovers from an acute appendicitis, with the appendix more or less damaged, he still has concretions or mucus inclusion which are very likely to make trouble subsequently, although in a way his statement is true that any attack is protective, but in the cases in which the appendix has undergone more or less destruction in an acute attack the scar tissue which remains does not contract so regularly upon remaining filaments, nerve filaments, and that is the difference. In the cases of fibroid degeneration of the appendix, cases of protective appendicitis, nerve filaments are regularly engaged in the sclerotic connective tissue, but in the scar tissue following an acute attack nerve filaments will be found to have been destroyed along with other structures. That covers most of the salient points, I believe.

The President: The chair recognises that the committee appointed a few moments ago have returned, and if there be no objection we will hear from Dr. Crego, the chairman of that committee.

Dr. Crego: Dr. O'Brien is the secretary of the committee, and as he has the report I will ask him to read it.

Dr. O'Brien read the report upon the death of Dr. Krauss.

It was moved and seconded that the report be accepted, and by a rising vote it was unanimously carried.

Dr. Creveling called for the reading of a paper entitled, "Appendicitis," by Dr. Albert L. Beahan, of Canandaigua.

Dr. Beahan: I feel that after sitting at the feet of a master of the profession that it is a little out of keeping to place this paper before you, but in a sense we workers in the country are sifters of the teachings and the work of those who are in the larger centers, and it may act as something of a reflection of their work to listen to one of the followers.

Dr. Creveling: The time is getting so short and the papers so numerous that the business committee feel compelled to ask to go over this discussion and pass to the next paper.

The President: If there be no objection the business committee will pass to the next paper.

Dr. Nathan Jacobson, of Syracuse, read a paper entitled, "Infantile Scurvy Involving the Hip Joint."

The President: The question has arisen, gentlemen, whether or no the constitution does not make provision for the selection of a second and a third vice-president. The chair is informed that the constitution does make provision for the selection of a second vice-president, and that there has evidently been an omission at some of your annual sessions, no selection having been made for that particular office. I therefore ask if the association desires to take any action upon this matter and of the omission that has been made heretofore.

Dr. Elsner: Is a nomination in order?

The President: If there be no objection we will return to the order of business.

Dr. Elsner: I nominate Dr. Faff of Oneida.

No other nominations were made.

Dr. Elsner moved that the secretary cast the ballot of the association for Dr. Faff of Oneida, for second vice-president.

The secretary cast the ballot of the association for Dr. Faff for second vice-president, and the president declared him duly elected second vice-president for the ensuing term.

Dr. Stephenson in the chair.

Dr. Martin B. Tinker of Ithaca, read a paper entitled, "Important Factors in Diagnosis and Treatment of Surgical Tuberculosis."

Dr. Henry L. Elsner, of Syracuse, read a paper entitled, "Treatment of High Blood Pressure and the Control of Arteriosclerosis."

Dr. A. A. Young, of Newark, read a paper entitled, "Ichthyosis."

Dr. Wynkoop, Syracuse: The hour is late and there are several papers remaining, and I move you that the remaining papers be read by title.

Dr. Creveling: That remains with the business committee.

Dr. Conway in the chair.

The President: The chair will have to rule that the business committee, having had the power delegated to them, is right in the position it takes.

Dr. Crego: I wish to make a motion thanking the Auburn members of the association for the nicely prepared program, whoever were the committee that prepared it, for the delicious luncheon served us, and the general efficiency of the order in general.

Dr. Young seconded the motion.

Carried.

Dr. Creveling: Mr. President, it is suggested that we return to the regular order of business.

The President: If there be no objection, there will be a return to the regular order of business.

Dr. Creveling: Mr. President, as you know, we have a small honorary list of members, members that have been very carefully selected, members who in some branch or other have risen to great prominence in the profession, and I move that Dr. Robert T. Morris be added to that list of our honorary members.

Motion seconded.

The President: Before that motion is put I would like to say, as your presiding officer, that I believe by this proposed action this association does itself honor. Personally I have known Dr. Morris for more than twenty-five years, and, like others of you, I have seen him rise in the surgical world, until today he attains the eminence that is conceded to him by every student of surgery in this country.

Motion carried.

The Secretary: For the past few years the county list of which this society is composed, and which is printed on the back of the program, has in a way excluded Tompkins county, which takes in Ithaca and the adjoining towns, and I move that Tompkins county be added to the counties included in the association.

Motion seconded and carried.

Dr. Creveling: Mr. President, your business committee believes it proper to dispense with the reading of any more papers, as Dr. Wynkoop says, it is getting late, and it is also an imposition to ask Dr. Wynkoop to read his paper before the meeting now with so many members absent, and your committee would not do so, and it is therefore suggested that we discontinue the reading of all papers, and that a motion to adjourn would be in order at any time.

The President: Prior to discontinuing the reading of the papers do you make a motion in reference to the papers being received by title?

Dr. Creveling: The papers will all be sent for publication just the same.

The President: A motion to that effect will be in order.

Dr. Creveling moved that all unread papers be received by the secretary by title and published in the proceedings.
Motion seconded and carried.

It was moved and seconded that the meeting adjourn.
Seconded and carried.

